# Outcomes of surgical treatment on type A acute aortic dissection accompanied with coronary artery involvement

**DOI:** 10.3389/fsurg.2022.950264

**Published:** 2022-09-26

**Authors:** Wei Qin, Rui Fan, Jiankai Wang, Jian Li, Fuhua Huang, Xin Chen

**Affiliations:** ^1^ Department of Thoracic and Cardiovascular Surgery, Nanjing First Hospital, Nanjing Medical University, Nanjing, China; ^2^ School of Medicine, Southeast University, Nanjing, China

**Keywords:** acute aortic dissection, coronary artery involvement, local coronary repair, coronary artery bypass grafting, surgical outcomes, survival rate

## Abstract

**Background:**

Coronary artery (CA) involvement due to acute aortic dissection (AAD) is a catastrophic cardiovascular disease with high mortality. Two main surgery strategies, local coronary repair and coronary artery bypass grafting (CABG) can be applied to reestablish the blood flow in the aortic repair. This study was to evaluate the operative and long-term outcomes of type A AAD patients, who received aortic dissection repair plus CABG or local coronary repair.

**Method:**

We reviewed our database and screened 148 type A AAD patients with CA involvement from January 2001 to December 2021. Local coronary repair or CABG was performed concomitantly on these enrolled patients.

**Results:**

At the time of aortic repair, there were 58 patients with concomitant CABG (Group I) and 90 patients with local coronary repair (Group II). The basal characteristics of these two groups had no difference, except for acute myocardial ischemia (AMI) and CA involvement type. 45 patients with AMI in Group I, but none in Group II (*P* < 0.001). There was a higher frequency of type B and C lesions of CA involvement in Group I than that in Group II (*P* < 0.001). There was no difference in surgical procedures and complications, except for postoperative acute kidney injury (AKI) (34.5% vs. 8.9%, *P* < 0.001). Hospital mortality in Group I was higher than that in Group II, but without statistical difference (20.7% vs. 11.1%, *P* = 0.155). No significant difference was obtained in long-term survival rate between the two groups (82.5 ± 4.8% vs. 81.2 ± 6.9%, *P* = 0.19).

**Conclusion:**

CABG and local coronary repair suits different types of CA involvement, and their effects on perioperative results and long-term survival for type A AAD patients with CA involvement are equal.

## Introduction

Coronary artery (CA) involvement due to acute aortic dissection (AAD) may mislead the correct diagnosis and is associated with high mortality resulting from acute heart failure (1, 2). According to the International Registry of Acute Aortic Dissection (IRAD), the preoperative ECG showed nonspecific ST and T-wave changes in 42%, ischemic changes in 15%, and evidence of an acute myocardial infarction in 5% of type A AAD (2). In most circumstance, although the coronary ostia are involved, there is no myocardial ischemia, so in fact the overall rate of type A AAD with CA involvement is higher than that diagnosed by preoperative ECG, reaching as high as 24.3% reported by Lu S and his colleagues in China (3).

Coronary artery bypass grafting (CABG) using saphenous vein (SV) is one of the most common and easiest strategies to re-establish an adequate coronary blood flow and rescue jeopardized myocardium in aortic dissection operations (3, 4). Conversely, some surgeons recommend direct local coronary repair for restoring physiologic antegrade blood flow; however, compared with CABG, local coronary repair is more complex and has higher requirements (5). There are few reports about surgical outcomes and long-term results of these patients who received different procedures on CA involvement. Therefore, in this present study, we review our database to analyze the perioperative results and long-term survival of type A AAD patients underwent aortic repair plus concomitant CABG or local coronary repair.

## Methods

### Study protocol and population

This study was approved by the ethical committee of Nanjing First Hospital (KY20190404-03-KS-01) and all patients provided their consent. From January 2001 to December 2021, 892 aortic dissection patients received aortic repair in our single center. We reviewed our database and found 154 patients accompanied with CA involvement in all. Among them, 1 patient with preoperative cerebral ischemia and 2 with lower extremity ischemia were excluded. Another 3 patients who did aortic repair plus coronary bypass due to concomitant coronary artery disease (CAD) were also excluded. Finally, the rest of 148 type A AAD patients were divided into two groups according to the different operations on CA reconstruction, including 58 aortic repair plus CABG (Group I) and 90 aortic repair plus local coronary repair (Group II). CA involvement was diagnosed clinically by the operating surgeons in cooperation and in review of clinical findings (computed tomography angiography imaging, electrocardiogram), and the intraoperative visualization of a torn/compression CA. All patients had preoperative CTA and intraoperative TEE to evaluate the true /false lumen, the entry tear, branch involvement, heart and valve function, etc. There was no patient who did coronary artery angiography prior to emergency dissection operations in all these 148 patients. The preoperative characteristics were listed in Table 1.

**Table 1:** Demographics and characteristics of patients.

Variables	Group I (*n* = 58)	Group II (*n* = 90)	*P*
Gender (male), *n*	38	64	0.474
Age, y	52.1 ± 10.5	50.6 ± 11.7	0.414
Hypertension, *n*	56	85	0.847
Diabetes, *n*	10	15	0.927
Smoking, *n*	35	52	0.757
Pericardial tamponade, *n*	2	3	1.0
Previous PCI, *n*	0	1	1.0
Prevoius renal dysfunction	1	1	1.0
Interval time from the onset of AAD to operation, h	15.0 ± 7.7	17.2 ± 9.1	0.139
Which side CA involved			
Isolated left, *n*	2	3	1.0
Isolated right, *n*	51	80	0.858
Bilateral, *n*	5	7	1.0
AMI, *n*	45	0	<0.001
Aortic stenosis, *n*	6	10	0.883
Aortic insufficiency (≧ moderate), *n*	3	5	1.0
Ascending aorta aneurysm, *n*	11	17	0.991
Aortic root aneurysm, *n*	6	4	0.289
CA involvement type			
Type A, *n*	4	88	<0.001
Type B, *n*	18	2	<0.001
Type C, *n*	36	0	<0.001

AAD, acute aortic dissection; CA, coronary artery; AMI, acute myocardial ischemia.

### Surgical procedures

#### Establishment of cardiopulmonary bypass

For all patients, median sternotomy was performed in general anesthesia. Cardiopulmonary bypass (CPB) and antegrade cerebral perfusion (ACP) were established as described previously (6). Right axillary artery and right atrium cannulation were routinely used to establish CPB. If the right axillary artery was unsuitable for cannulation, the femoral artery was substituted, and brachiocephalic artery was perfused directly for ACP during circulatory arrest. A left ventricular sump was inserted through the pulmonary vein to decompress the ventricle. In case of unstable hemodynamics or cardiac shock due to severe pericardial tamponade, the femoral artery and femoral vein were used for cannulation and initiation of CPB before sternotomy.

#### Strategies on CA involvement

After cross-clamping the aorta, the aortic sinus and the CA ostium were fully exposed and carefully examined. According to the classification from Neri and his colleagues (5), our strategies on CA involvement were as follows:

(1) Cardioplegia was delivered through the left and right CA ostium directly in patients with type A or B coronary artery involvement. When occlusion or avulsion of the CA was present (type C), we always use a special plastic needle inserted into the coronary ostium to deliver cardioplegia firstly while the other surgeons harvest SV rapidly. And then a SV graft was anastomosed to the CA rapidly in a standard manner. More cardioplegia was then infused through the reversed SV bypass before proximal and distal aortic repair.
(2) Local coronary repair was done in patients with type A lesions and CABG was underwent in patients with type B or C lesions.
(3) In another situation, CABG was a rescue method in some patients during operation when TEE indicated the heart dysfunction due to the unsatisfied CA repair.
(4) The proximal end of the SV was anastomosed directly to the ascending vascular graft or the innominate limb of the four-branched vascular graft.
(5) There was no internal mammary artery bypass placed on the affected coronary vessel in this study.

#### Aortic repair procedures

Patients were aimed to cool to a nasopharyngeal temperature of approximately 24 °C and rectal temperature of 27 °C *via* CPB. During the cooling process, the proximal aortic surgery was done, including aortic valve repair /replacement, Bentall procedure, partial aortic root remodeling and ascending aorta replacement etc. Aortic repair was similar to the past publications (7, 8). Partial root reconstruction means one or two aortic sinus was replaced by artificial patch owing to the dissection, which was described in our past report (9). CPB was discontinued when the nasopharyngeal temperature reached 25 °C (hypothermia circulatory arrest, HCA). The brain continued to be perfused at a rate of approximately 5∼10 ml/kg.min through the right axillary artery cannulation during HCA. The distal aortic repair included isolated ascending, hemi-arch, or total arch replacement depending on the pathology of the aortic arch (tear location, aortic diameter, etc). Aortic arch operation was performed in HCA with ACP and open distal anastomosis.

### Follow-up protocol

The standard follow-up protocols for these discharged patients were as follows: performing aorta enhanced CT at least once a year; having a telephone interview or outpatient interview at least once a year; doing coronary artery CTA at least once during follow-up time in CABG patients.

## Statistical analysis

Categorical variables were expressed as percentages, and continuous variables were expressed as mean ± standard deviation with range. Statistical analysis was performed by Student's t-test, if variances were not equal (tested by Leven's test), Mann-Whitney-U-Test was performed. Chi-squared test (Fisher exact tests if *n *≤ 5) was used for categorical variables. Survival analysis was performed according to the methods of Kaplan-Meier, and statistical differences were analyzed using the log-rank test. All statistical analysis was performed with SPSS 13.0 software. All *P*-values less than 0.05 were considered statistically significant.

## Results

### Preoperative data

The preoperative variables of the two groups were listed in Table 1. The primary analysis revealed that there was no statistical difference in gender, age, hypertension, diabetes, smoking, PCI history, pericardial tamponade, previous renal dysfunction, aortic insufficiency /stenosis, aortic aneurysm between the two groups. The interval time from the onset of AAD to operation also did not differ in the two groups. However, 45 patients in Group I suffered acute myocardial ischemia (AMI) while no patient in Group II had AMI (*P* < 0.001). The right CA was much more often affected than the left CA. Bilateral CA involvement was present in 5 and 7 patients in Group I and II, respectively. Furthermore, CA involvement type was totally different between the two groups. Obviously, there was a higher frequency of type B and C lesions of CA involvement in Group I than that in Group II (*P* < 0.001).

### Operative details

In Group I, CABG was done firstly before aortic repair in 54 patients with type B or C lesions. In addition, CABG acted as a rescue procedure in 4 patients (all belong to type A involvement) who had local coronary repair at first. For these 4 patients, when we were trying to discontinue CPB and found the right ventricle dysfunction, so SV bypass was put to right coronary artery (RCA) for each of them. One graft was needed in 53 patients, two grafts in 3 and three grafts in 2. RCA was the only revascularized vessel in 51 cases, left coronary artery (LCA) alone in 2, and both coronary systems in 5. In Group II, although there were 2 patients with type B lesions, we repaired the right coronary ostia and did Bentall procedure successfully. Details on the surgical procedures of the proximal and distal aortic repair were summarized in Table 2. No significant difference can be seen in these proximal and distal procedures.

**Table 2:** Details of the proximal and distal aortic repair.

Procedures	Group I (*n* = 58)	Group II (*n* = 90)	*P*
Proximal			–
STJ anastomosis, *n*	35	56	0.819
AVR, *n*	7	11	0.978
AVP, *n*	5	5	0.697
Bentall, *n*	6	10	0.883
Partial root reconstruction, *n*	5	7	1.0
MVP, *n*	1	2	1.0
Distal			–
Isolated ascending aorta, *n*	1	1	1.0
Hemi-arch, *n*	19	24	0.462
Total arch + elephant trunk, *n*	38	65	0.387
CABG (graft to)			
RCA, *n*	51		
LCA, *n*	2	–	–
RCA + LCA, *n*	5		

STJ, sinutubular junction; AVR, aortic valve replacement; AVP, aortic valve plasty; MVP, mitral valve plasty.

### Surgical outcomes

Operative outcomes were reported in Table 3. The analysis indicated the CPB time, circulatory arrest time, ICU stay time, and in-hospital time did not differ between the two groups, except for the cross-clamp time (136.5 ± 35.1 min vs. 119.4 ± 22.7 min; *P* < 0.001). Patients in Group I had a higher occurrence of postoperative acute kidney injury (AKI) than that in Group II (34.5% vs. 8.9%, *P* < 0.001). But statistical difference was not found in the other major complications. Details were as follows: new stroke, 4 in Group I vs. 3 in Group II (*P* = 0.548); new paraplegia, 2 vs. 1 (*P* = 0.698); tracheotomy, 6 vs. 4 (*P* = 0.196); ventilation more than 4 days, 8 vs. 7 (*P* = 0.272); and gastrointestinal bleeding, 3 vs. 2 (*P* = 0.614). One patient in Group I could not discontinue the CPB and required extracorporeal membrane oxygenation (ECMO). Additionally, in Group I, two patients underwent ascending-femoral artery bypass and 1 did femoral-femoral artery bypass surgery concomitantly because lower extremity hypotension after aortic surgery. In Group II, 3 patients did ascending-femoral artery bypass and 1 did femoral-femoral artery bypass owing to the same reason. Twelve hospital deaths (20.7%) were observed in Group I: 4 died of heart failure and multiple organ dysfunction syndrome (MODS), 4 died of severe infection, 1 died of AKI, 1 died of visceral ischemia, and 2 patients in fatal cerebral edema died of giving up further treatment. By contrast, 10 patients (11.1%) in Group II died in hospital: 3 attributed to MODS, 2 attributed to fatal cerebral infarction, 4 attributed to severe infection, and 1 attributed to intestinal ischemia. The hospital mortality of Group I was higher than that of Group II, but Fisher's exact test showed no statistical difference between the two groups (20.7% vs. 11.1%, *P* = 0.155).

**Table 3:** The overview of surgical outcomes.

Operative variables	Group I (*n* = 58)	Group II (*n* = 90)	*P*
CPB time, min	210.9 ± 63.7	198 ± 33.3	0.109
Cross-clamp time, min	136.5 ± 35.1	119.4 ± 22.7	<0.001
Circulatory arrest time, min	21.8 ± 5.3	22.6 ± 3.5	0.286
ICU stay time, d	5.8 ± 5.8	4.7 ± 3.0	0.125
In-hospital time, d	20.4 ± 10.8	19.1 ± 7.4	0.388
Postoperative complications			–
AKI, *n*	20	8	<0.001
New stroke, *n*	4	3	0.548
Paraplegia, *n*	2	1	0.698
Tracheotomy, *n*	6	4	0.196
Ventilation ≧4 days, *n*	8	7	0.272
GI bleeding, *n*	3	2	0.614
Re-explore for bleeding, *n*	1	1	1.0
ECMO supporting, n	1	0	1.0
Cause of death in hospital	12	10	–
Fatal cerebral infarction, *n*	2	2	–
Severe infection, *n*	4	4	–
AKI, *n*	1	0	–
MODS, *n*	4	3	–
Visceral ischemia, *n*	1	1	
Hospital mortality, %	20.7	11.1	0.155

CPB, cardiopulmonary bypass; ICU, intensive care unit; AKI,acute kidney injury; GI, gastrointestinal; MODS, multiple organ dysfunction syndrome; ECMO, extracorporeal membrane oxygenation.

### Follow-up and survival rate

**Figure 1:**
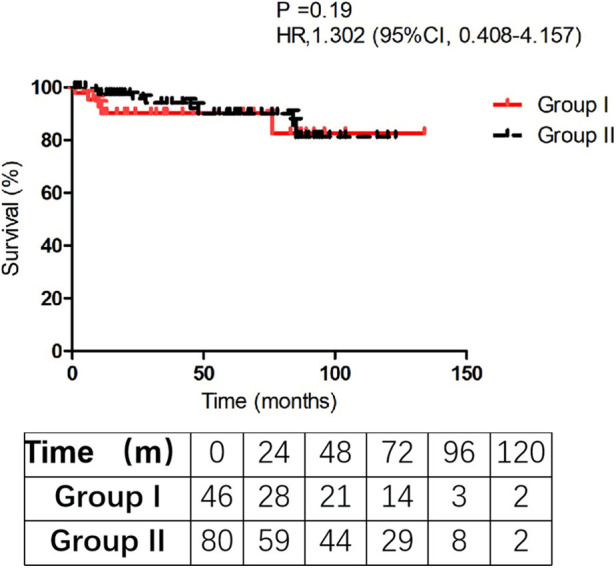
Survival rate of the two groups during the follow-up.

Follow-up was successfully obtained in 116 discharged patients (116/126, 92.1%) with a mean follow-up time (44.3 ± 35.8) and (54.5 ± 32.8) months in Group I and II, respectively (*P* = 0.108). Five patients died in Group I and 8 patients in Group II died during the follow-up. In Group I, one died of lung infection, 1 died of heart failure, 1 died of renal dysfunction, 1 died of cerebral hemorrhage, and 1 died without clear reason. In Group II, 2 died of cerebral stroke, 2 died of heart dysfunction, 1 died of GI cancer, and 3 died with unknown causes. The Kaplan-Meier analysis estimated that 10-year survival rate was 82.5 ± 4.8% in Group I vs. 81.2 ± 6.9% in Group II (*P* = 0.19). We failed to find a significant difference in survival rate between the two groups in the follow-up (Figure 1). The analysis indicated that concomitant CABG in aortic dissection repair patients did not increase the long-term mortality.

### Patency of SV bypass

**Figure 2:**
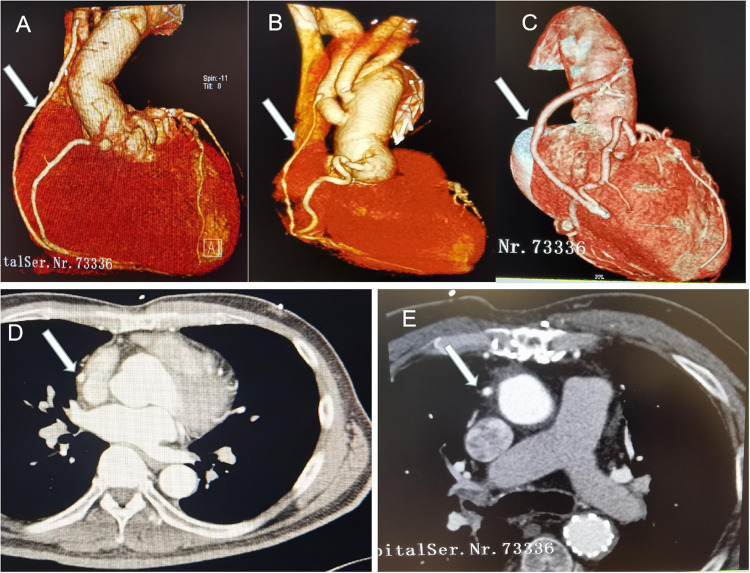
CT pictures in the follow-up time. Arrows indicate the SV bypass. (**A**) SV to RCA bypass+ hemi-arch replacement; (**B**) SV to PDA bypass + total arch replacement, SV bypass with obvious stenosis; (**C**) SV to right acute marginal artery bypass +hemi-arch replacement; (**C,D**) CT images of the SV bypass.

In Group I, forty-two patients (42/46, 91.3%) had CTA scan of the coronary artery during the follow-up period. CTA results showed 100% patency rate of the SV bypass, no matter which coronary artery performed (Figure 2). However, SV bypass with obvious stenosis can be seen in 2 patients but who did not complain chest pain or dyspnea (see Figure 2B). Noticeably, native coronary arteries which did CABG were not occlusive.

## Discussion

The incidence of acute myocardial ischemia due to type A AAD has been reported at 5.7% to 11.3% (5, 10). However, the overall rate of type A AAD with CA involvement in our study is as high as 17.3% (154/892), which is higher than the incidence of myocardial ischemia reported in the past reports. Lien and his colleagues (11) reported there were 264 patients underwent surgery for type A AAD during 1997 to 2017, 34 (12.9%) of them had CA involvement identified during surgery. But, the number of patients with myocardial ischemia in that study was 21 (21/34, 61.8%). Also, Eren et al. (10), Kawahito et al. (12), and Imoto et al. (13) presented their incidence of myocardial ischemia in CA involvement patients were 78.6% (11/14), 75.0% (9/12), and 64.0% (48/75), respectively. In this study, there were 45 patients in Group I who suffered myocardial ischemia in a total of 148 patients. So, the prevalence of myocardial ischemia in CA involvement patients was 30.4% (45/148), which is much lower than the past reports. Conceptually, it is generally assumed that CA involvement results in coronary ischemia. Nonetheless, 103 (69.6%) patients in our present study showed no evidence of coronary ischemia despite CA involvement existed. Actually, it is not very easy to evaluate CA involvement preoperatively. Some factors, such as location, the primary entry, flow pattern in the false lumen, and the interval time from the onset of AAD to operation may impact CA involvement. Furthermore, the condition of the false lumen may change dramatically after onset of the aortic dissection. That is to say, AAD extending to the coronary ostia may keep stable and it also may cause myocardial ischemia at any time. Thus, we think the description of “CA involvement” might be a more appropriate term than “coronary malperfusion” for this special cohort.

Although some reports (14–16) from China and abroad present satisfactory surgical results with a mortality rate less than 10% in type A AAD patients undergoing aortic repair, CA involvement due to type A AAD still carries a higher operative mortality. We regard that rapid restoration of myocardial perfusion and correction of the damaged aortic root structure is essential to prevent prolonged myocardial ischemia. However, the controversy that still exists regarding the ideal method of restoring blood flow to the malperfused myocardium.

Regarding surgical strategy, Neri and his colleagues (5) concluded that type A lesions can be directly repaired, type B and type C lesions can be treated by local coronary repair or CABG. They described various local repair techniques and preferred repair of dissected coronary arteries over CABG. However, Eren (10) and Kawahito (12) never performed local coronary repair or re-implantation because they believed that mobilization and repair of acutely dissected coronary arteries was potentially dangerous and difficult. CABG was relatively simple and well-controlled, which was regarded to use safely in all types of coronary artery dissection in their clinic. Zhang and his colleagues (17) retrospectively investigated the results of 489 patients with type A AAD and concluded that CABG was suitable for type B and C lesions and local repair was only for type A, which is consistent with many other reports (3, 5, 18). From our experience, it is reasonable and maneuverable to follow these surgical principles: type A, ostium local repair or reimplantation; type B, CABG if repair fails; type C, CABG. In this study, we can see type B and C lesions accounted for 93.1% in Group I and type A lesions accounted for 97.8% in Group II. So, we did CABG in Group I and local repair in Group II. Encountering shortness of repair experience, we think CABG is a better option because it not only save time but also can ensure that the seriously damaged or even completely occluded coronary arteries of patients are unobstructed again.

Additionally, some others (19, 20) recommended urgent emergency percutaneous coronary intervention (PCI) and stent the dissected coronary artery before starting to prepare the operating room. We think it is a good solution to restore coronary flow for some type A AAD patients with unstable hemodynamics. But, it does not solve the aortic dissection and still carries high risk of aortic rupture and cardiac tamponade. Furthermore, these studies are only case reports, there was no big data evidence. So, I think timely surgical treatment is still the first choice for these patients.

In a study of consecutive 382 patients with type A AAD in Switzerland, Morjan et al. (21) identified concomitant CABG as a predictor of in-hospital mortality [Odds Ratio (OR) = 3.8115, 95% CI = 0.514–2.138, *P* = 0.001], and found concomitant CABG was associated with high hospital mortality. Many other reports (10, 12, 17, 18) also demonstrated that concomitant CABG carries a significant operative risk in type A AAD patients undergoing surgical aortic repair. In the current study, we could not get the statistical difference probably owing to the small sample size. But, we can obviously see the tendency of higher operative mortality in concomitant CABG group. Actually, I think it is the myocardial ischemia rather than CABG leads to the high mortality in such patients. In Table 1, we can see 45 patients (77.6%) had myocardial ischemia in Group I while none in Group II. Meanwhile, some authors (22, 23) argued that concomitant CABG was not a risk factor for hospital morality with aortic repair. Okada et al. (23) thought patients with concomitant CABG have more preoperative comorbidities, which may adversely affect outcomes, and which may therefore deserve special attention.

Patel et al. (24) suggested that immediate reperfusion and timely surgical repair should be performed for type A AAD patients with malperfusion. They found that those patients who survive the initial malperfusion and undergo aortic repair have a similar late survival when compared with those patients presenting with uncomplicated dissection. Zhang and his colleagues (17) retrospectively analyzed 21 patients who received concomitant CABG at the same time of aortic repair in type A AAD and found that these survivors may benefit from concomitant CABG and had similar midterm mortality compared with the other cases. As shown in Figure 1, the two curves are relatively parallel during the follow-up time. Although this mortality rate of concomitant CABG group was worse than that in patients without CABG, once these patients were discharged from the hospital, the survival rate during follow-up was satisfactory (*P* = 0.19).

## Limitation

Firstly, this is a single-center retrospective study with a small sample. Secondly, the two groups have the different baseline characteristics, especially in acute myocardial ischemia which may adversely affect outcomes. Thirdly, follow-up material was not detailed. We have to accumulate more cases and more detailed information to evaluate the treatment.

## Conclusion

CABG is simple, less invasive, and well-controlled technique, which is more suitable for type B and C lesions. Local coronary repair may be the optimal choice for type A patients. Both concomitant CABG and local coronary repair at the time of aortic repair in type A AAD patients have the same midterm survival rate.

## Data Availability

The raw data supporting the conclusions of this article will be made available by the authors, without undue reservation.
